# Combined exposure to Maneb and Paraquat alters transcriptional regulation of neurogenesis-related genes in mice models of Parkinson’s disease

**DOI:** 10.1186/1750-1326-7-49

**Published:** 2012-09-28

**Authors:** Paula Desplats, Pruthul Patel, Kori Kosberg, Michael Mante, Christina Patrick, Edward Rockenstein, Masayo Fujita, Makoto Hashimoto, Eliezer Masliah

**Affiliations:** 1 Department of Neuroscience, University of California San Diego, 9500 Gilman Dr., MTF 344, La Jolla, CA, 92093-0624, USA; 2 Department of Pathology, University of California San Diego, 9500 Gilman Dr., La Jolla, CA, 92093-0624, USA; 3 Laboratory for Chemistry and Metabolism, Tokyo Metropolitan Institute for Neuroscience, Tokyo, 183-8526, Japan

**Keywords:** Adult neurogenesis, Parkinson’s disease, Maneb, Paraquat, Pesticides, Environmental exposure, Gene expression, Gene x environment interactions

## Abstract

**Background:**

Parkinson's disease (PD) is a multifactorial disease where environmental factors act on genetically predisposed individuals. Although only 5% of PD manifestations are associated with specific mutations, majority of PD cases are of idiopathic origin, where environment plays a prominent role. Concurrent exposure to Paraquat (PQ) and Maneb (MB) in rural workers increases the risk for PD and exposure of adult mice to MB/PQ results in dopamine fiber loss and decreased locomotor activity. While PD is characterized by neuronal loss in the substantia nigra, we previously showed that accumulation of α-synuclein in the limbic system contributes to neurodegeneration by interfering with adult neurogenesis.

**Results:**

We investigated the effect of pesticides on adult hippocampal neurogenesis in two transgenic models: Line 61, expressing the human wild type *SNCA* gene and Line LRRK2(G2019S), expressing the human *LRRK2* gene with the mutation G2019S. Combined exposure to MB/PQ resulted in significant reduction of neuronal precursors and proliferating cells in non-transgenic animals, and this effect was increased in transgenic mice, in particular for Line 61, suggesting that α-synuclein accumulation and environmental toxins have a synergistic effect. We further investigated the transcription of 84 genes with direct function on neurogenesis. Overexpresion of α-synuclein resulted in the downregulation of 12% of target genes, most of which were functionally related to cell differentiation, while LRRK2 mutation had a minor impact on gene expression. MB/PQ also affected transcription in non-transgenic backgrounds, but when transgenic mice were exposed to the pesticides, profound alterations in gene expression affecting 27% of the studied targets were observed in both transgenic lines. Gene enrichment analysis showed that 1:3 of those genes were under the regulation of FoxF2 and FoxO3A, suggesting a primary role of these proteins in the response to genetic and environmental cues.

**Conclusions:**

We report that adult neurogenesis is highly susceptible to multiple “risk factors” for PD, including α-synuclein accumulation, LRRK2 G2019 mutation and exposure to environmental toxins. We identified specific groups of genes that are responsive to each stressor, while uncovering a novel function for Fox transcription factors in PD.

## Background

Parkinson's disease (PD) is the second most common neurodegenerative disorder in the elderly, affecting 2% of the population over 60 years old [1]. The classic form of PD is manifested clinically by resting tremor and postural instability, along with variable non-motor symptoms. Neuropathologically, PD is characterized by the loss of dopaminergic neurons mainly in the substantia nigra pars compacta accompanied by the formation of intracytoplasmic inclusions known as Lewy bodies, containing α-synuclein. Neurodegeneration in PD affects primarily the striatonigral system, but cases with cognitive impairment present more widespread degeneration including neuronal populations in the striatum, hippocampus, and neocortex [2,3]. Therefore, PD associated pathology not only has an impact on the degeneration of mature dopaminergic neurons in the basal ganglia, but could also influence neurogenesis in the adult brain [4], where neural stem and progenitor cells are still present in the hippocampal dentate gyrus and the subventricular zone (SVZ), and continuously generate new neurons [5-7].

From etiology, PD is believed to be a multifactorial disease where environmental factors might act on genetically predisposed individuals [8]. Only a small fraction of PD occurrence (about 5 % of cases) are inherited in a recessive or dominant manner and are associated with mutations in genes including *SNCA, LRRK2, DJ1, PINK1and UCHL-1*[9,10]. Although mutations in the *SNCA* gene (encoding for α-synuclein) are rare, the crucial roles of α-synuclein in PD pathology, including aberrant calcium homoeostasis and mitochondrial fragmentation, is supported by multiple neuropathological and biochemical evidence.

Mutations in the same genes can be involved in familial PD and also be risk factors for sporadic manifestations; suggesting that inherited and idiopathic PD share common pathological mechanisms [11]. While accumulation/missfolding of α-synuclein might play more prominent roles in PD sporadic manifestations, mutations in the *LRRK2* gene, encoding leucine-rich repeat kinase 2, are the most prevalent cause of autosomal dominantly inherited PD, which are characterized by brainstem Lewy body pathology. The most frequent mutation, LRRK2(G2019S) is located in the kinase domain of the protein increasing kinase activity [12] and has the highest genotype- and population-attributable risk [13].

We have recently shown that accumulation of α-synuclein in the limbic system might contribute to the neurodegenerative phenotype by interfering with adult neurogenesis in transgenic mice models [14,15]. We reported reduced proliferation and neuronal maturation accompanied by increased apoptosis in murine embryonic stem (mES) cells overexpressing wild type and mutant α-synuclein, and in the hippocampal subgranular zone of α-synuclein transgenic mice. These alterations were accompanied by a reduction in Notch-1 and Hairy and Hes-5 mRNA and protein levels [16].

LRRK2 protein, on the other hand, shows widespread, neuronal-specific expression in the adult mammalian brain, and is highly expressed in the hippocampus and subventricular zone (SVZ) [17], suggesting its role in neurogenesis. LRKK2 have been recently implicated in modulation of neuronal differentiation in murine embryonic stem cells [18]. Moreover, adult neurogenesis and neurite outgrowth have been reported to be impaired in LRKK2(G2019S) mice [19].

Besides genetic-linked manifestations, the majority of PD cases are of idiopathic origin, whose etiology is yet not completely understood. Recent studies however, suggest that interactions between environmental toxins and genetic polymorphisms might play a role. Neurotoxins, including agrichemicals might lead to neurodegeneration by triggering accumulation of α-synuclein in subcortical and cortical regions [20]. Concurrent exposure to the herbicide Paraquat (PQ) and the fungicide Maneb (MB) in adult mice led to significantly dopamine (DA) fiber loss, altered DA turnover and decreased locomotor activity [21,22]. Moreover, combined exposure to MB and PQ in rural workers was reported to increase the risk of developing PD by 75% in agricultural areas of California [23]. PQ is one the most used herbicides worldwide. PQ exerts its toxicity by cellular redox cycling with the formation of superoxide radicals and it is believed that mitochondrial complex I is a primary target [24]. MB, used as a fungicide, seems to cross the brain blood barrier and, although its mechanisms of toxicity are not very well known, it seems to preferentially inhibit mitochondrial complex III [8].

Although extensive research has been conducted on the effects of pesticides on the dopaminergic system in the PD brain, a possible impact of pesticide exposure on adult neurogenesis remained to be explored.

We extended here our previous studies on adult hippocampal neurogenesis [4,14-16,19] in two different transgenic mice mouse models of PD generated in our laboratory, the Line 61, expressing the human wild type *SNCA* gene and Line 29 that expresses LRRK2(G2019S), by investigating the effects of MB and PQ exposure and with the aim to model gene x environment interactions in familial and sporadic PD manifestations. Our findings show that α-synuclein missfoldig/accumulation, LRRK2 gene mutation G2019S and exposure to the agrichemicals Maneb and Paraquat have deleterious effects on adult neurogenesis, contributing to overall PD pathology.

## Results and discusion

The mThy1- α-synuclein tg (Line 61) expresses high levels of human α-synuclein throughout the brain, including the basal ganglia and the substantia nigra [25]. This model reproduces some aspects of PD, with progressive changes in dopamine release and striatal content, α-synuclein pathology and deficits in motor and non-motor functions [26]. The mThy1-LRRK2(G2019S) transgenic mice present a 3 fold overexpression of the transgene in the neocortex (Additional File 1).

To analyze the effects of fungicides/herbicides on adult neurogenesis, transgenic animals of both lines and their non-transgenic littermate controls were administered i.p. injections of Maneb (15 mg/kg) and Paraquat (5 mg/kg) or saline (for control groups), twice a week during 21 days. We centered our study on the adult neurogenic niche in the SVZ by immunohistochemical detection of neurogenesis markers and by the focused analysis of the expression of 84 genes related to neurogenesis and neural stem cell physiology by real time-PCR-arrays.

**Figure 1:**
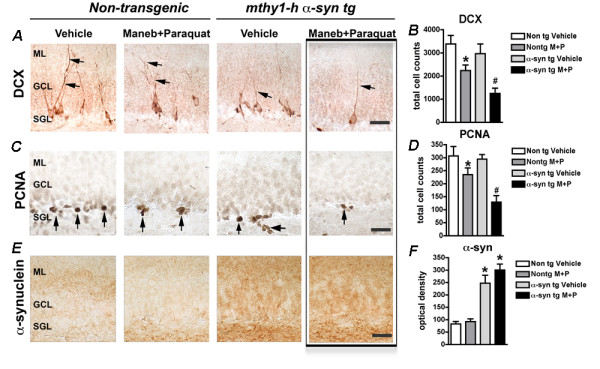
**Exposure to Maneb andParaquat alters adult neurogenesisin the hippocampus ofα-Synuclein transgenic mice*.*** Immunohistochemical detection of Doublecortin (DCX) positive neuronal precursors (**A**) and Proliferating cell nuclear antigen (PCNA) positive proliferating cells (**C**) in hippocampus of non-transgenic or Line 61 mice overexpressing human α-synuclein (**E**). Image analysis showing total counts of DCX (**B**) and PCNA (**D**) immunoreactive cells. (**F**) Optical density analysis showing accumulation of α-synuclein transgenic mice. Bar represents 10 μm. One way ANOVA or Student’s *t* test was used to determine statistical significance, *, # p<0.05.

**Figure 2:**
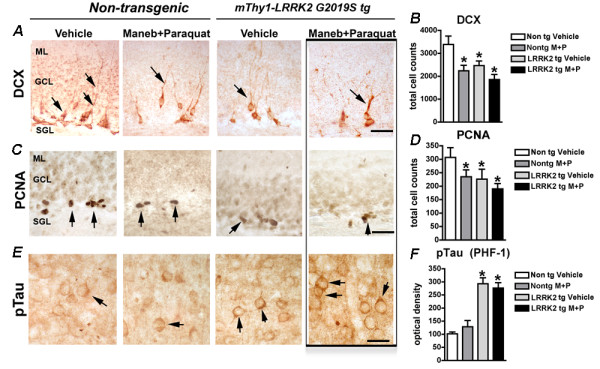
**Exposure to Maneb andParaquat alters adult neurogenesisin the hippocampus ofLRRK2(G2019S) transgenic mice*.*** Immunohistochemical detection of Doublecortin (DCX) positive neuronal precursors (**A**) and Proliferating cell nuclear antigen (PCNA) positive proliferating cells (**C**) in hippocampus of non-transgenic or LRRK2(G2019S) mice. Image analysis showing total counts of DCX (**B**) and PCNA (**D**) immunoreactive cells. (**E**-**F**) Immunodetection and optical density analysis showing accumulation of phosphorylated Tau characteristic of LRRK2(G2019S) transgenic mice. Bar represents 10 μm. One way ANOVA or Student’s *t* test was used to determine statistical significance, *, # p<0.05.

We first evaluated the effects of MB/PQ exposure on adult neurogenesis at the cellular level by immunohistochemical detection of neuronal precursors and proliferating cells in the hippocampus of non-transgenic and transgenic animals with or without exposure to MB/PQ (Figures 1 and 2). Combined exposure to agrichemicals resulted in a significant reduction in neuronal precursors (DCX positive) and proliferating cells (PCNA positive) in non-transgenic animals (Figure 1A-D and Figure 2A-D). While overexpression of α-synuclein in Line 61 transgenic animals had minimal effects on neurogenesis (Figure 1A-D), exposure of these transgenic animals to MB/PQ resulted in a significant loss of DCX and PCNA positive cells (Figure1A-D) suggesting that α-synuclein accumulation and environmental toxins have a synergistic effect. On the other hand, analysis of LRRK2 (G2019S) transgenic mice revealed that the mutation impacts adult neurogenesis by reduction of neuronal precursors and proliferating cells (Figure 2A-D), but these alterations were only slightly increased by subsequent exposure to MB/PQ (Figure 2A-D).

We next investigated if transcriptional disregulation might underlie the observed alterations in neurogenesis. To analyze the contribution of each genetic background, we first compared gene expression profiles between transgenic animals and their non-transgenic littermates only treated with saline (Table 1). In agreement with our previous findings, overexpresion of α-synuclein resulted in the downregulation of 10 genes of our target panel (~12%), most of which were functionally related to cell differentiation and migration (Table 1A). Overexpression of LRRK2(G2019S), on the other hand, had a minor impact on neurogenesis-related gene expression, with only 3 genes been disregulated (<4%), without functional relation (Table 1B).

**Table 1:** **Genetic Effects: genes dysregulatedin PD transgenic micemodels**

**Accession**	**Fold change**	**Gene title**	**Gene symbol**	**Functional Group**
***(A) mThy-α-synuclein transgenic (Line61)***				
NM_145990	−1.56	CDK5 regulatory subunit associated protein 2	Cdk5rap2	Cell differentiation
NM_008711	−1.44	Noggin	Nog	
NM_008380	−1.34	Inhibin beta-A	Inhba	
NM_010894	−1.26	Neurogenic differentiation 1	Neurod1	
NM_009170	−1.21	Sonic hedgehog	Shh	
NM_008744	−1.33	Netrin 1	Ntn1	Cell motility and migration
NM_021409	−1.30	Par-6 (partitioning defective 6) homolog beta (C. elegans)	Pard6b	
NM_010275	−1.46	Glial cell line derived neurotrophic factor	Gdnf	Growth factors
NM_013904	−1.26	Hairy/enhancer-of-split related with YRPW motif 2	Hey2	Transcription
NM_007889	−1.24	Dishevelled 3, dsh homolog (Drosophila)	Dvl3	Cell signaling
***(B) mThy-LRRK2 mut G2019S(Line G2019S)***				
NM_145990	1.58	CDK5 regulatory subunit associated protein 2	Cdk5rap2	Cell differentiation
NM_009630	−1.27	Adenosine A2a receptor	Adora2a	Cell signaling, apoptosis
NM_022312	−1.24	Tenascin R	Tnr	Cell adhesion

Comparison of gene expression between each of the transgenic lines studied with their corresponding non-transgenic littermates treated with saline. P<0.05 per Student’s *t* test.

We further evaluated if adult neurogenesis could be a direct target for environmental toxins by comparing gene expression levels between non transgenic animals exposed to MB/PQ to those treated with saline (Table 2). Exposure to agrichemicals resulted in the altered transcription of another set of 10 genes, half of which overlapped with the genes disregulated in the transgenic models, suggesting that environmental and genetic conditions share common molecular targets. We therefore analyzed how gene x environment interactions might potentiate their individual effects on adult neurogenesis. Comparison of transcriptomes between MB/PQ treated transgenic animals and non-transgenic saline controls revealed profound alterations in gene expression for both lines (Tables 3 and 4). We found that 27% of the screened genes (23 out of 84) were downregulated in Line 61 animals (Table 3) and this time, a similar extent of disregulation was observed for LRRK2(G2019S) mice treated with MB/PQ, with 22 out of 84 transcripts being significantly altered (Table 4). As previously observed for the pesticide exposure, cell differentiation was the most affected functional group, followed by the expression of growth factors, transcriptional regulation, synaptic function and cell adhesion. Only 50% of the altered transcripts were also affected by either genetic or environmental factors independently, with the remaining 50% of targets being specifically altered in response to gene x environment interactions.

**Table 2:** **Environmental effects: genes dysregulatedin response to MB/PQ**

**Accession**	**Fold change**	**Gene title**	**Gene symbol**	**Functional Group**
NM_007488	1.25	Aryl hydrocarbon receptor nuclear translocator 2	Arnt2	Transcription
NM_194053	1.22	Reticulon 4	Rtn4	Apoptosis
NM_145990	1.24	CDK5 regulatory subunit associated protein 2	Cdk5rap2	Cell differentiation
NM_008380	−1.54	Inhibin beta-A	Inhba	
NM_008711	−1.27	Noggin	Nog	
NM_010894	−1.23	Neurogenic differentiation 1	Neurod1	
NM_008744	−1.40	Netrin 1	Ntn1	Cell motility
NM_007540	−1.35	Brain derived neurotrophic factor	Bdnf	Growth factors
NM_022312	−1.23	Tenascin R	Tnr	Cell adhesion
NM_174991	−1.21	Brain-specific angiogenesis inhibitor 1	Bai1	Cell proliferation

Comparison of gene expression between non-transgenic animals treated with Maneb/Paraquat with non-transgenic animals treated with saline as control group. P<0.05 per Student’s *t* test.

**Table 3:** **Gene x Environment Interaction:genes altered by MB/PQin α-syn-Tg**

**Accession**	**Fold change**	**Gene title**	**Gene symbol**	**Functional Group**
NM_145990	−2.35	CDK5 regulatory subunit associated protein 2	***Cdk5rap2***	Cell differentiation
NM_007865	−1.82	Delta-like 1 (Drosophila)	Dll1	
NM_008380	−1.80	Inhibin beta-A	***Inhba***	
NM_008711	−1.68	Noggin	***Nog***	
NM_010894	−1.33	Neurogenic differentiation 1	***Neurod1***	
NM_009170	−1.33	Sonic hedgehog	***Shh***	
NM_008553	−1.26	Achaete-scute complex homolog 1 (Drosophila)	Ascl1	
NM_008782	−1.24	Paired box gene 5	Pax5	
NM_010275	−1.25	Glial cell line derived neurotrophic factor	***Gdnf***	Growth factors
NM_009505	−1.33	Vascular endothelial growth factor A	***Vegfa***	
NM_007553	−1.28	Bone morphogenetic protein 2	Bmp2	
NM_007554	−1.25	Bone morphogenetic protein 4	***Bmp4***	
NM_010077	−1.31	Dopamine receptor D2	Drd2	Synaptic transmission
NM_013503	−1.23	Dopamine receptor D5	Drd5	
NM_013904	−1.64	Hairy/enhancer-of-split related with YRPW motif 2	***Hey2***	Transcription
NM_013905	−1.61	Hairy/enhancer-of-split related with YRPW motif-like	Heyl	
NM_007889	−1.52	Dishevelled 3, dsh homolog (Drosophila)	***Dvl3***	Cell signaling
NM_001008533	−1.30	Adenosine A1 receptor	Adora1	
NM_008744	−1.39	Netrin 1	***Ntn1***	Cell motility
NM_174991	−1.28	Brain-specific angiogenesis inhibitor 1	***Bai1***	Cell proliferation
NM_022312	−1.27	Tenascin R	***Tnr***	Cell adhesion
NM_013660	−1.26	Sema domain, Ig domain, transmembrane domain and short cytoplasmic domain, 4D	Semad4	Apoptosis
NM_009685	−1.38	Amyloid beta (A4) precursor protein-binding, family B,1	Apbb1	Cell cycle

Comparison of gene expression between mThy-α-synuclein transgenic mice treated with Maneb/Paraquat, with their corresponding non-transgenic littermates treated with saline. P<0.05 per Student’s *t* test. Bold italic fonts denote genes also affected by genetic background or pesticides independently.

**Table 4:** **Gene x Environment Interaction:genes altered by MB/PQin LRRK2-Tg**

**Accession**	**Fold Change**	**Gene title**	**Gene symbol**	**Functional Group**
NM_145990	1.32	CDK5 regulatory subunit associated protein 2	***Cdk5rap2***	Cell differentiation
NM_178591	−1.37	Neuregulin 1	Nrg1	
NM_025876	−1.32	CDK5 regulatory subunit associated protein 1	Cdk5rap1	
NM_008380	−1.31	Inhibin beta-A	***Inhba***	
NM_013627	−1.29	Paired box gene 6	Pax6	
NM_010894	−1.26	Neurogenic differentiation 1	***Neurod1***	
NM_009170	−1.24	Sonic hedgehog	***Shh***	
NM_009871	−1.22	CDK5 regulatory subunit 1 (p35)	Cdk5r1	
NM_007540	−1.24	Brain derived neurotrophic factor	***Bdnf***	Growth factors
NM_009505	−1.23	Vascular endothelial growth factor A	***Vegfa***	
NM_007554	−1.23	Bone morphogenetic protein 4	***Bmp4***	
NM_013904	−1.30	Hairy/enhancer-of-split related with YRPW motif 2	***Hey2***	Transcription
NM_008900	−1.26	POU domain, class 3, transcription factor 3	Pou3f3	
NM_007488	−1.24	Aryl hydrocarbon receptor nuclear translocator 2	***Artn2***	
NM_022312	−1.27	Tenascin R	***Tnr***	Cell adhesion
NM_010110	−1.21	Ephrin B1	Efnb1	
NM_183171	−1.21	Fasciculation and elongation protein zeta 1 (zygin I)	Fez1	
NM_174991	−1.37	Brain-specific angiogenesis inhibitor 1	***Bai1***	Cell proliferation
NM_010883	−1.21	Norrie disease (pseudoglioma)	Ndph	
NM_203491	−1.24	Cholinergic receptor, muscarinic 2	Chrm2	Synaptic functions
NM_009599	−1.21	Acetylcholinesterase	Ache	
NM_008744	−1.39	Netrin 1	***Ntn1***	Cell motility and migration

Comparison of gene expression between mThy-LRRK2(G2019) transgenic mice treated with Maneb/Paraquat, with their corresponding non-transgenic littermates with saline. P<0.05 per Student’s *t* test. Bold italic fonts denote genes also affected by genetic background or pesticides independently.

**Figure 3:**
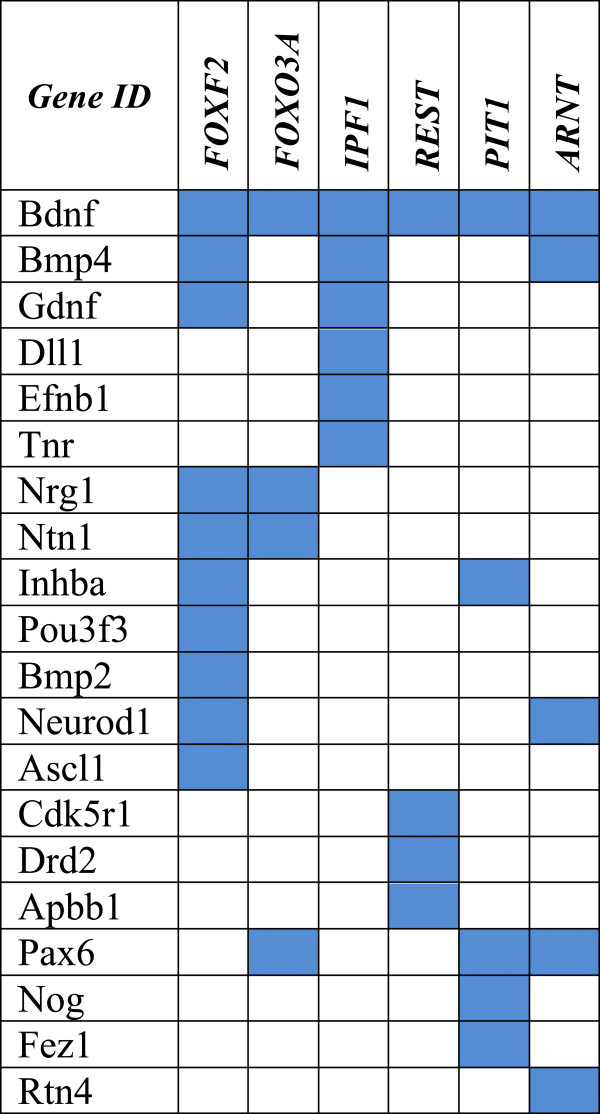
**Gene Set Enrichment analysisof disregulated transcripts*.*** Gene Set Enrichment analysis was performed using the Molecular Signature Database “motif-based” to search for known and likely regulatory elements present in the promoters and 3’ UTR on our entire group of 38 affected genes. Shadow boxes represent positive hits for the regulatory proteins on the top row for each tested gene in the first column with a p<0.001. *FOXF2:* forkhead box F2; *FOXO3A:* forkhead box O3A; *IPF1:* Insulin promoter factor 1; *REST:* RE1-silencing transcription factor; *PIT1:* POU domain, class1, transcription factor 1 (Pit1) and *ARNT:* aryl hydrocarbon receptor nuclear translocator.

To determine if common regulatory factors might mediate this broad transcriptional disregulation we performed Gene Set Enrichment analysis using the Molecular Signature Database category C3, “motif-based” to search for known and likely regulatory elements present in the promoters and 3’ UTR on our entire group of 38 affected genes [27]. From the input list, 34 genes (>89%) were catalogued sequences that entered the analysis. Significant enrichment was obtained for FoxF2 that was present in 10 genes (p<0.001), followed but IPF1, FoxO3A, PIT1, ARNT and REST (Figure 3). We obtained positive hits for Fox family members in 12 genes, almost 1:3 of the altered sequences, suggesting a primary role of these regulatory proteins in mediating the response of neuronal progenitors to genetic and environmental cues. 

## Conclusions

In conclusion, we report here that adult neurogenesis is highly susceptible to multiple “risk factors” for PD, including α-synuclein accumulation, LRRK2 G2019S mutation and exposure to environmental toxins, namely the fungicide Maneb and the pesticide Paraquat. Each of these three factors, either alone or in combination, extensively affect the expression of genes that regulate stem cell proliferation, fate determination, neuronal differentiation and survival. Cell differentiation and the expression of growth factors needed to sustain the new neuronal lineage appear to be the most affected steps in neurogenesis, thus aberrant transcription might mediate reduced survival and integration of newborn neurons into the existing circuitries. Our current results extend previous studies from our group and others showing that neurogenesis is reduced in the olfactory bulb and the hippocampus of adult α-synuclein transgenic mice by diminished survival of neuronal precursors [14] and, importantly, that the neurogenic system is compromised in human PD brains, where proliferation of neural progenitor cells is decreased in the hippocampus and SVZ [28].

**Figure 4:**
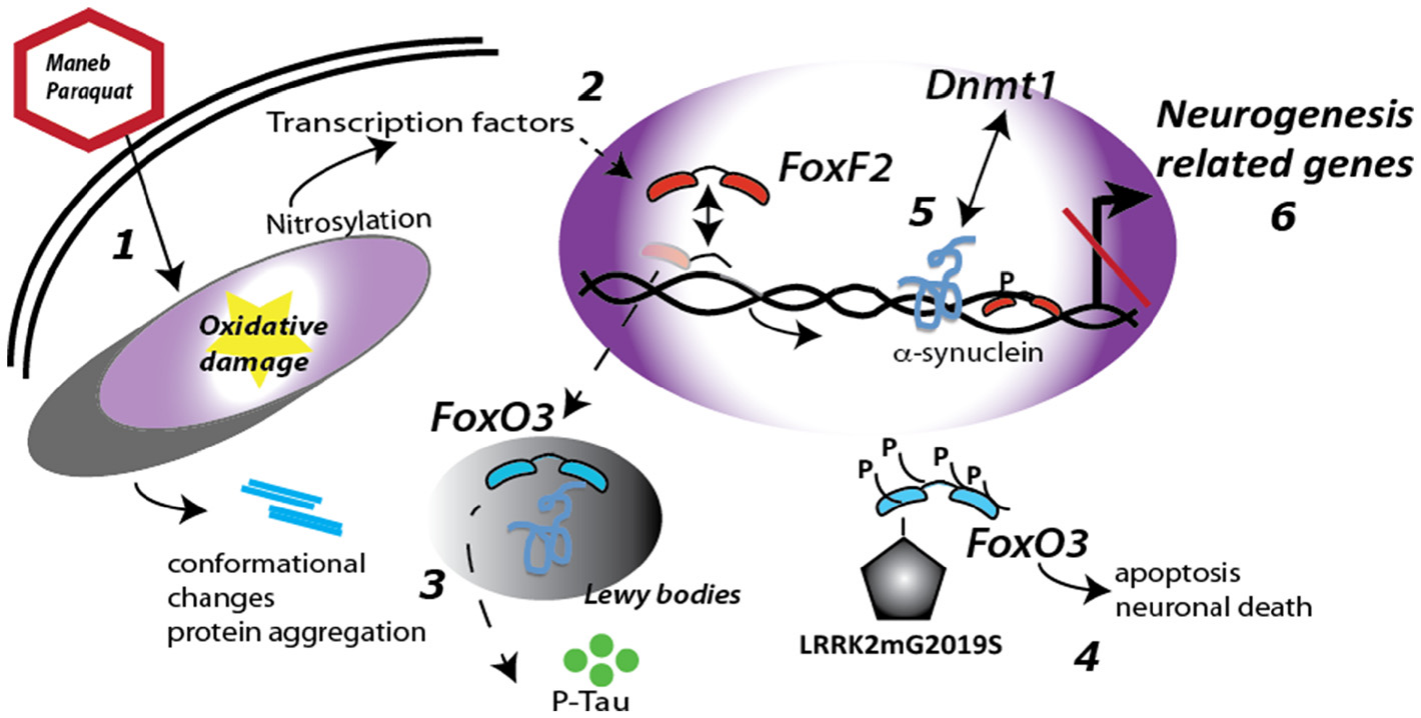
**Hypothetical model linking geneand environmental factors totranscriptional disregulation of neurogenesis-relatedgenes*.*** Maneb and Paraquat trigger ROS production (**1**), nitrosylation of transcription factors (**2**), and missfolding and aggregation of proteins, like α-synuclein. Lewy body formation in association with α-synuclein aggregation and phosphorylated-Tau accumulation in the context of LRRK2 mutations might contribute to misslocalization of Fox proteins (**3**) or aberrant phosphorylation triggering apoptosis and neuronal death (**4**). Alpha-synuclein can directly bind DNA and also alter DNA methylation (**5**). The combination of these factors will impact on the transcription of Fox-regulated neurogenesis-related genes (**6**).

We were able to identify specific groups of genes that are responsive to genetic variability (α-synuclein accumulation or LRKK2 G2019S mutation); to environmental toxins (MB/PQ exposure) or to the combination of genetic and environmental factors. Moreover, gene set enrichment analysis uncovered a novel function for members of the Fox family of transcription factors in PD. Forkhead box (Fox) transcription factors belong to a large family of regulatory proteins known to be involved in embryonic development, cell proliferation and differentiation [29,30]. Different lines of evidence suggest the involvement of particular Fox proteins in PD pathology: LRRK2 activity is needed for phosphorylation-dependent activation of FoxO [31] and ectopic localization of FoxO3a associated with Lewy bodies has been reported in human postmortem PD brains [32]. We propose a model integrating well-characterized molecular pathways leading to PD (Figure 4) in which MB and PQ interfere with mitochondria triggering oxidative stress and ROS production (1), which contribute to the nitrosylation and inactivation of transcription factors [33] (2), and missfolding and aggregation of proteins, like α-synuclein. Lewy body formation in association with α-synuclein aggregation and phosphorylated-Tau accumulation in the context of LRRK2 mutations might also contribute to misslocalization of Fox proteins (3) or aberrant phosphorylation triggering apoptosis and neuronal death (4). In addition, α-synuclein can directly bind to GC rich DNA regions abundant in promoters [34] and also alters DNA methylation [35] (5). The combination of these factors will impact on the transcription of Fox-regulated neurogenesis-related genes and result in reduced adult neurogenesis (6). 

## Methods

### Animal models

Experiments were conducted on 10-month old-transgenic mice expressing either wild type human *SNCA* (*mthy1-h α-syn tg*) or human LRRK2 harboring the G2019S mutation (*mthy1-h LRRK2 mut G2019*) under the Thy-1 promoter [25]; and on age and sex matched non-transgenic (C57/B6) control animals (n=8/group). Animals were injected intraperitoneally with saline or a sequential combination of Paraquat (5 mg/kg; Sigma) followed by Maneb (15 mg/kg; Sigma) twice a week for 21 days. Injections were applied 2 hours apart from each other. Brains were extracted after cervical dislocation and immediately frozen or fixed. All animals were handled in strict accordance with good animal practice and all procedures were completed under the specifications set forth by the UCSD Institutional Animal Care and Use Committee.

### Immunohistochemical analysis of neurogenesis markers

Briefly, as described previously [14,15], free-floating sections from mouse brains were treated with 0.6% H_2_O_2_ in Tris-buffered saline (TBS) (0.15 M NaCl and 0.1 M Tris- HCl, pH 7.5) for 30 min and incubated with antibodies against proliferating cell nuclear antigen (PCNA) (mouse monoclonal, 1:250; Santa Cruz Biotechnology), doublecortin (DCX) (goat polyclonal, 1:100; Santa Cruz Biotechnology), α-synuclein (rabbit polyclonal, 1:500; Millipore Bioscience) or mouse monoclonal antibody against phosphorylated Tau (1:250, a kind gift from Dr. Peter Davies at Albert Einstein, School of Medicine, NYC) overnight at 4°C. Sections were incubated for 1 h with the corresponding biotinylated secondary antibodies. After rinses in TBS, avidin– biotin–peroxidase complex was applied for 1 h, and then peroxidase detection was performed for 10 min (25 mg/ml diaminobenzidine, 0.01% H2O2, and 0.04% NiCl in TBS).

### Real-time PCR analysis

Total RNA was extracted from mice brains using the RNeasy mini kit (Qiagen, Valencia, CA) and was reverse transcribed using RT^2^ First Strand kit (SA Biosciences, Frederick, MD) from 1 μg of total RNA. Real-time PCR analysis was performed with the RT^2^ Profiler PCR Array for mouse Neurogenesis and Neural Stem Cells (PAMM-404 SABiosciences) according to the manufacturer’s instructions and using the StepOnePlus^TM^ real-time PCR system (Applied Biosystems, Carlsbad, CA). PCR reactions were performed on four independent sets of templates. Relative quantification of gene expression for the 84 gene targets present in the array was achieved by RT^2^ Profiler PCR Array Data Analysis version 3.5 (SABiosciences) using β-actin as an internal control. Additional normalization was achieved by feeding the expression values of a panel of “stem cell genes” including Sox2 and Nestin (stem cell/proliferation cell marker) and DCX and N-CAM (neuronal progenitor cells markers) into the software to account for specific cell loss observed in neurogenic cells. Quantitative analysis of gene expression was performed in n=4/6 animals/group. Genes were considered as disregulated if they presented a statistical significant change of 1.2 fold in expression compared to controls (p<0.05).

### Statistical analysis

Data represents mean values ± SEM from at least three independent experiments. Statistical analysis was performed using One-way ANOVA followed by the Tukey’s multiple comparison test or Student’s *t* test (unpaired; two-tailed) with a significance of *p*<0.05 (Prism Graph Pad Software).

## Competing interests

The authors declare that they have no competing interests.

## Authors' contributions

PD and EM conceived the study, participated in its design and wrote the manuscript. PP and KK carried out the real-time PCR array experiments. MM and ER carried out the animal experimentation. ER, MF and MH generated the LRRK2 transgenic mouse model. CP carried out the immunoassays. PD coordinated the whole study. All authors read and approved the final manuscript.

## Supplementary Material

Additional file 1 **Desplats.** Generation of mThy1-LRRK2 (G2019S) transgenic mice model. Click here for file

## References

[B1] Dauer W Przedborski S Parkinson's disease: mechanisms and models Neuron 2003 39 6 889 909 12971891 10.1016/s0896-6273(03)00568-3

[B2] Harding AJ Lakay B Halliday GM Selective hippocampal neuron loss in dementia with Lewy bodies Ann Neurol 2002 51 1 125 128 11782993 10.1002/ana.10071

[B3] McKeith I Mintzer J Aarsland D Burn D Chiu H Cohen-Mansfield J Dickson D Dubois B Duda JE Feldman H Dementia with Lewy bodies Lancet Neurol 2004 3 1 19 28 14693108 10.1016/s1474-4422(03)00619-7

[B4] Winner B Desplats P Hagl C Klucken J Aigner R Ploetz S Laemke J Karl A Aigner L Masliah E Dopamine receptor activation promotes adult neurogenesis in an acute Parkinson model Exp Neurol 2009 219 2 543 552 19619535 10.1016/j.expneurol.2009.07.013 PMC5038985

[B5] Carleton A Petreanu LT Lansford R Alvarez-Buylla A Lledo PM Becoming a new neuron in the adult olfactory bulb Nat Neurosci 2003 6 5 507 518 12704391 10.1038/nn1048

[B6] Eriksson PS Perfilieva E Bjork-Eriksson T Alborn AM Nordborg C Peterson DA Gage FH Neurogenesis in the adult human hippocampus Nat Med 1998 4 11 1313 1317 9809557 10.1038/3305

[B7] Sanai N Tramontin AD Quinones-Hinojosa A Barbaro NM Gupta N Kunwar S Lawton MT McDermott MW Parsa AT Manuel-Garcia Verdugo J Unique astrocyte ribbon in adult human brain contains neural stem cells but lacks chain migration Nature 2004 427 6976 740 744 14973487 10.1038/nature02301

[B8] Cicchetti F Drouin-Ouellet J Gross RE Environmental toxins and Parkinson's disease: what have we learned from pesticide-induced animal models? Trends Pharmacol Sci 2009 30 9 475 483 19729209 10.1016/j.tips.2009.06.005

[B9] Martin I Dawson VL Dawson TM Recent advances in the genetics of Parkinson's disease Annu Rev Genomics Hum Genet 2011 12 301 325 21639795 10.1146/annurev-genom-082410-101440 PMC4120236

[B10] Tan EK Skipper LM Pathogenic mutations in Parkinson disease Hum Mutat 2007 28 7 641 653 17385668 10.1002/humu.20507

[B11] Cookson MR The role of leucine-rich repeat kinase 2 (LRRK2) in Parkinson's disease Nat Rev Neurosci 2010 11 12 791 797 21088684 10.1038/nrn2935 PMC4662256

[B12] West AB Moore DJ Biskup S Bugayenko A Smith WW Ross CA Dawson VL Dawson TM Parkinson's disease-associated mutations in leucine-rich repeat kinase 2 augment kinase activity Proc Natl Acad Sci U S A 2005 102 46 16842 16847 16269541 10.1073/pnas.0507360102 PMC1283829

[B13] Healy DG Falchi M O'Sullivan SS Bonifati V Durr A Bressman S Brice A Aasly J Zabetian CP Goldwurm S Phenotype, genotype, and worldwide genetic penetrance of LRRK2-associated Parkinson's disease: a case–control study Lancet Neurol 2008 7 7 583 590 18539534 10.1016/S1474-4422(08)70117-0 PMC2832754

[B14] Winner B Lie DC Rockenstein E Aigner R Aigner L Masliah E Kuhn HG Winkler J Human wild-type alpha-synuclein impairs neurogenesis J Neuropathol Exp Neurol 2004 63 11 1155 1166 15581183 10.1093/jnen/63.11.1155

[B15] Winner B Rockenstein E Lie DC Aigner R Mante M Bogdahn U Couillard-Despres S Masliah E Winkler J Mutant alpha-synuclein exacerbates age-related decrease of neurogenesis Neurobiol Aging 2008 29 6 913 925 17275140 10.1016/j.neurobiolaging.2006.12.016 PMC2896275

[B16] Crews L Mizuno H Desplats P Rockenstein E Adame A Patrick C Winner B Winkler J Masliah E Alpha-synuclein alters Notch-1 expression and neurogenesis in mouse embryonic stem cells and in the hippocampus of transgenic mice J Neurosci 2008 28 16 4250 4260 18417705 10.1523/JNEUROSCI.0066-08.2008 PMC2666311

[B17] Melrose HL Kent CB Taylor JP Dachsel JC Hinkle KM Lincoln SJ Mok SS Culvenor JG Masters CL Tyndall GM A comparative analysis of leucine-rich repeat kinase 2 (Lrrk2) expression in mouse brain and Lewy body disease Neuroscience 2007 147 4 1047 1058 17611037 10.1016/j.neuroscience.2007.05.027

[B18] Schulz C Paus M Frey K Schmid R Kohl Z Mennerich D Winkler J Gillardon F Leucine-rich repeat kinase 2 modulates retinoic acid-induced neuronal differentiation of murine embryonic stem cells PLoS One 2011 6 6 e20820 21695257 10.1371/journal.pone.0020820 PMC3111438

[B19] Winner B Melrose HL Zhao C Hinkle KM Yue M Kent C Braithwaite AT Ogholikhan S Aigner R Winkler J Adult neurogenesis and neurite outgrowth are impaired in LRRK2 G2019S mice Neurobiol Dis 2011 41 3 706 716 21168496 10.1016/j.nbd.2010.12.008 PMC3059106

[B20] Wills J Credle J Oaks AW Duka V Lee JH Jones J Sidhu A Paraquat, but not maneb, induces synucleinopathy and tauopathy in striata of mice through inhibition of proteasomal and autophagic pathways PLoS One 2012 7 1 e30745 22292029 10.1371/journal.pone.0030745 PMC3264632

[B21] Thiruchelvam M Brockel BJ Richfield EK Baggs RB Cory-Slechta DA Potentiated and preferential effects of combined paraquat and maneb on nigrostriatal dopamine systems: environmental risk factors for Parkinson's disease? Brain Res 2000 873 2 225 234 10930548 10.1016/s0006-8993(00)02496-3

[B22] Thiruchelvam M Richfield EK Baggs RB Tank AW Cory-Slechta DA The nigrostriatal dopaminergic system as a preferential target of repeated exposures to combined paraquat and maneb: implications for Parkinson's disease J Neurosci 2000 20 24 9207 9214 11124998 10.1523/JNEUROSCI.20-24-09207.2000 PMC6773035

[B23] Costello S Cockburn M Bronstein J Zhang X Ritz B Parkinson's disease and residential exposure to maneb and paraquat from agricultural applications in the central valley of California Am J Epidemiol 2009 169 8 919 926 19270050 10.1093/aje/kwp006 PMC2727231

[B24] Berry C La Vecchia C Nicotera P Paraquat and Parkinson's disease Cell Death Differ 2010 17 7 1115 1125 20094060 10.1038/cdd.2009.217

[B25] Rockenstein E Mallory M Hashimoto M Song D Shults CW Lang I Masliah E Differential neuropathological alterations in transgenic mice expressing alpha-synuclein from the platelet-derived growth factor and Thy-1 promoters J Neurosci Res 2002 68 5 568 578 12111846 10.1002/jnr.10231

[B26] Chesselet MF Richter F Zhu C Magen I Watson MB Subramaniam SR A Progressive Mouse Model of Parkinson's Disease: The Thy1-aSyn ("Line 61") Mice Neurotherapeutics 2012 10.1007/s13311-012-0104-2 PMC3337020 22350713

[B27] Xie X Lu J Kulbokas EJ Golub TR Mootha V Lindblad-Toh K Lander ES Kellis M Systematic discovery of regulatory motifs in human promoters and 3' UTRs by comparison of several mammals Nature 2005 434 7031 338 345 15735639 10.1038/nature03441 PMC2923337

[B28] Hoglinger GU Rizk P Muriel MP Duyckaerts C Oertel WH Caille I Hirsch EC Dopamine depletion impairs precursor cell proliferation in Parkinson disease Nat Neurosci 2004 7 7 726 735 15195095 10.1038/nn1265

[B29] Tuteja G Kaestner KH Forkhead transcription factors II Cell 2007 131 1 192 17923097 10.1016/j.cell.2007.09.016

[B30] Tuteja G Kaestner KH SnapShot: forkhead transcription factors I Cell 2007 130 6 1160 17889656 10.1016/j.cell.2007.09.005

[B31] Kanao T Venderova K Park DS Unterman T Lu B Imai Y Activation of FoxO by LRRK2 induces expression of proapoptotic proteins and alters survival of postmitotic dopaminergic neuron in Drosophila Hum Mol Genet 2010 19 19 3747 3758 20624856 10.1093/hmg/ddq289

[B32] Su B Liu H Wang X Chen SG Siedlak SL Kondo E Choi R Takeda A Castellani RJ Perry G Ectopic localization of FOXO3a protein in Lewy bodies in Lewy body dementia and Parkinson's disease Mol Neurodegener 2009 4 32 19627592 10.1186/1750-1326-4-32 PMC2723103

[B33] Nakamura T Lipton SA Redox modulation by S-nitrosylation contributes to protein misfolding, mitochondrial dynamics, and neuronal synaptic damage in neurodegenerative diseases Cell Death Differ 2011 18 9 1478 1486 21597461 10.1038/cdd.2011.65 PMC3178424

[B34] Hegde ML Rao KS DNA induces folding in alpha-synuclein: understanding the mechanism using chaperone property of osmolytes Arch Biochem Biophys 2007 464 1 57 69 17537399 10.1016/j.abb.2007.03.042

[B35] Desplats P Spencer B Coffee E Patel P Michael S Patrick C Adame A Rockenstein E Masliah E Alpha-synuclein sequesters Dnmt1 from the nucleus: a novel mechanism for epigenetic alterations in Lewy body diseases J Biol Chem 2011 286 11 9031 9037 21296890 10.1074/jbc.C110.212589 PMC3059002

