# Editorial Expression of Concern: Combined exposure to Maneb and Paraquat alters transcriptional regulation of neurogenesis-related genes in mice models of Parkinson’s disease

**DOI:** 10.1186/s13024-026-01012-5

**Published:** 2026-11-10

**Authors:** Molecular Neurodegeneration Editorial Board

**Affiliations:** The Campus, 4 Crinan Street, London, N1 9XW UK

**Editorial Expression of Concern: Molecular Neurodegeneration Editorial Board 7:49 (2012)**

**https://doi.org/10.1186/1750-1326-7-49**

**Correction**

The Editors-in-Chief would like to alert the readers that concerns have been raised regarding areas of high similarity in Fig. 1C Non-transgenic Vehicle group image.

In addition, Fig. 2C *mthy1-h α-syn tg* Manen+Paraquat image appears to overlap with Fig. 4F in the authors’ earlier article [1], which has been addressed by a correction [2].

The authors have been unable to provide the underlying raw data due to the age of the article. Readers are therefore advised to intepret these data with caution.

Paula Desplats and Elezier Masliah agree with this Editorial Expression of Concern. Edward Rockenstein is deceased. The other authors did not respond to any correspondence from the Publisher about this Editorial Expression of Concern.

## References

[1] Ubhi K, Inglis C, Mante M, Patrick C, Adame A, Spencer B, et al. Fluoxetine ameliorates behavioral and neuropathological deficits in a transgenic model mouse of α-synucleinopathy. Exp Neurol. 2012;234(2):405–416. 10.1016/j.expneurol.2012.01.008. PMC3897235 22281106

[2] Ubhi K, Inglis C, Mante M, Patrick C, Adame A, Spencer B, et al. Corrigendum to “Fluoxetine ameliorates behavioral and neuropathological deficits in a transgenic model mouse of α-synucleinopathy”Exp Neurol. 2012 April; 234(2): 405-416. Exp Neurol. 2024;379:114873. 10.1016/j.expneurol.2024.114873. 38937165

