# Supplementary material for: Combined exposure to Maneb and Paraquat alters transcriptional regulation of neurogenesis-related genes in mice models of Parkinson’s disease
Source: Mol Neurodegener. 2012 Sep 28;7:49. doi: 10.1186/1750-1326-7-49 (PMC3502617; doi:10.1186/1750-1326-7-49)

Additional File 1. Generation of mThy1-LRRK2 (G2019S) transgenic mice model. **(A)** Diagrammatic representation of the transgene in the mThy1 cassette and PCR analysis showing the corresponding fragments. **(B)** Real time PCR analysis showing the levels of mRNA expression of the human LRRK2 in line 29, bearing the G2019S mutation. **(C)** Western blot analysis in membrane and cytosolic fractions showing increased expression of the LRRK2 protein in the transgenic mouse compared to non-tg littermates. **(D)** Immunocytochemical analysis for LRRK2 displaying the patterns of expression of hLRRK2 in the control and transgenic mice. Bar represents 10 μm.

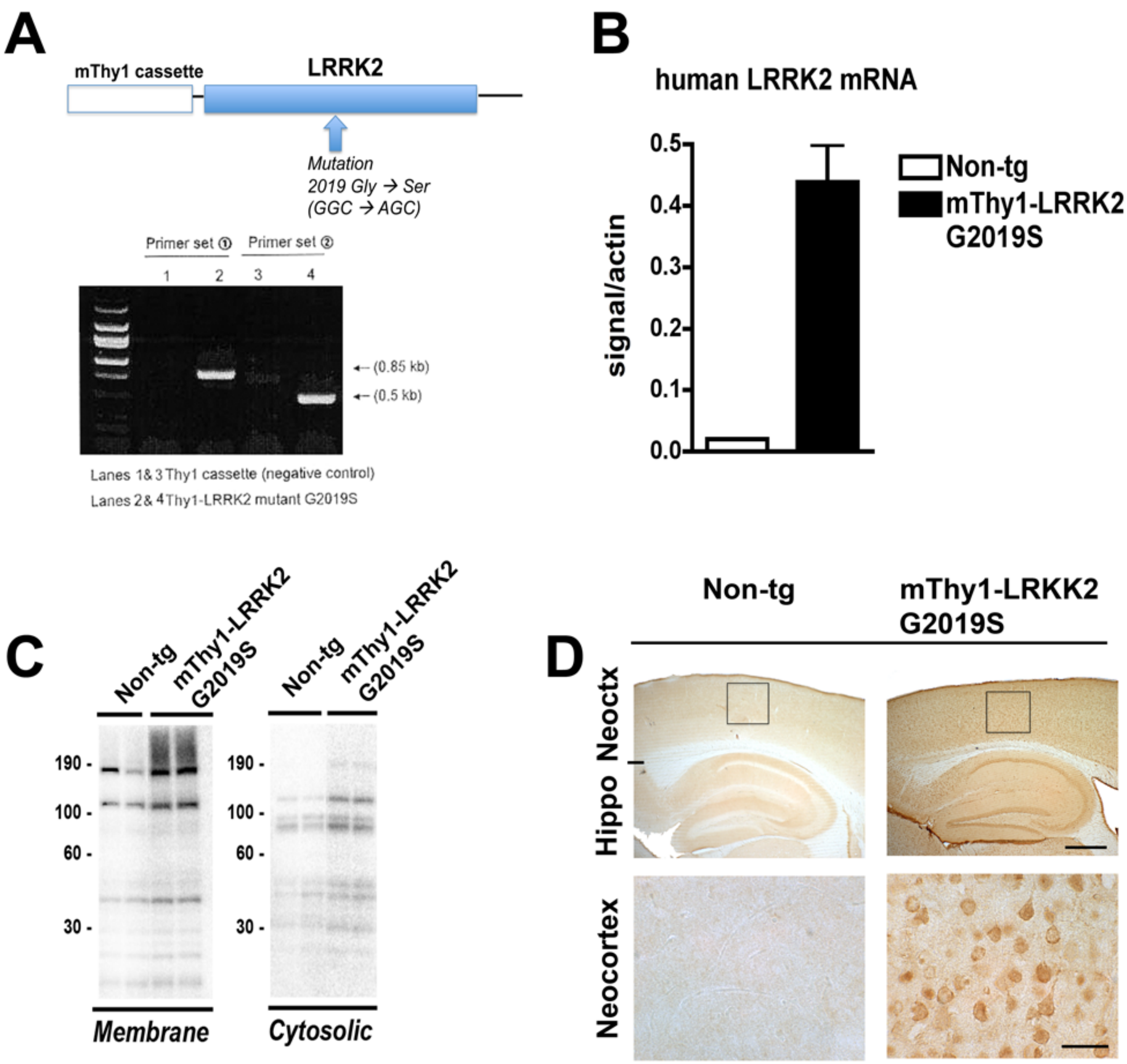

Supplement: Additional file 1 — Desplats. Generation of mThy1-LRRK2 (G2019S) transgenic mice model. [file 1750-1326-7-49-S1.pdf]
